# Atrial Tachycardias Arising from Ablation of Atrial Fibrillation: A Proarrhythmic Bump or an Antiarrhythmic Turn?

**DOI:** 10.4061/2010/950763

**Published:** 2010-04-07

**Authors:** Ashok J. Shah, Amir Jadidi, Xingpeng Liu, Shinsuke Miyazaki, Andrei Forclaz, Isabelle Nault, Lena Rivard, Nick Linton, Olivier Xhaet, Nicolas Derval, Frederic Sacher, Pierre Bordachar, Philippe Ritter, Meleze Hocini, Pierre Jais, Michel Haissaguerre

**Affiliations:** Hôpital Cardiologique du Haut-Lévêque, The Université Bordeaux II, 33604 Bordeaux-Pessac, France

## Abstract

The occurrence of atrial tachycardias (AT) is a direct function of the volume of atrial tissue ablated in the patients with atrial fibrillation (AF). Thus, the incidence of AT is highest in persistent AF patients undergoing stepwise ablation using the strategic combination of pulmonary vein isolation, electrogram based ablation and left atrial linear ablation. Using deductive mapping strategy, AT can be divided into three clinical categories viz. the macroreentry, the focal and the newly described localized reentry all of which are amenable to catheter ablation with success rate of 95%. Perimitral, roof dependent and cavotricuspid isthmus dependent AT involve large reentrant circuits which can be successfully ablated at the left mitral isthmus, left atrial roof and tricuspid isthmus respectively. Complete bidirectional block across the sites of linear ablation is a necessary endpoint. Focal and localized reentrant AT commonly originate from but are not limited to the septum, posteroinferior left atrium, venous ostia, base of the left atrial appendage and left mitral isthmus and they respond quickly to focal ablation. AT not only represents ablation-induced proarrhythmia but also forms a bridge between AF and sinus rhythm in longstanding AF patients treated successfully with catheter ablation.

## 1. Introduction

Atrial fibrillation (AF) is no longer a formidable rhythm since ablationists challenged this notorious arrhythmia more than a decade ago in their unprecedented quest for sinus rhythm (SR) [1]. Ablation strategies are based on clinical types of AF but nevertheless, the volume of tissue ablated to treat AF is highest for any cardiac arrhythmia described so far. Paroxysmal AF is amenable to catheter ablation with minimum atrial tissue destruction such that electrical isolation of pulmonary veins (PVs) suffices for establishing cure [2]. Persistent and longer lasting forms of AF necessitate extensive atrial tissue ablation in addition to PV isolation to restore SR [3–7]. Besides having evolved as a therapeutic option in symptomatic AF, surgical ablation has become a routine adjunct to many valvular surgeries and may be employed with surgical coronary revascularization and also as a “standalone” procedure [8, 9]. Despite improvements in ablation strategies, relatively high volume of tissue ablation is performed in AF. Together with remodeling of atria, it provides a favourable substrate for the development of sustained atrial tachycardia(s) during and after AF ablation (ATp) [4].

## 2. Magnitude of ATp Burden

Based on our observation and also that of others, the likelihood of occurrence of ATp is probably a function of the volume of tissue ablated for AF. Importantly, recent expansion of AF ablation has caused a surge in the incidence of ATp. Incidence of ATp after PV isolation has been variably reported from 2.9% to 10% [10–14]. Notably, circumferential PV isolation is associated with a significantly higher incidence of ATp than segmental isolation [12, 13]. In patients undergoing circumferential PV isolation and linear ablation, incidence of ATp has been reported to vary from >10% to 30% [15–18]. Nademanee et al. reported <10% patients developing ATp after isolated ablation of complex fractionated signals in AF wherein other groups have observed higher incidence. In a study on the effectiveness of ablation of complex fractionated signals without PV isolation and linear ablation in persistent AF performed in five centers, up to 40% patients were found to develop intraprocedural ATp (unpublished data). The incidence of ATp rises to 40% to 57% in patients wherein electrogram based atrial ablation accompanies PV isolation and linear ablation [3, 7, 19].

## 3. Classification

The Working Group Report classified atrial flutter/tachycardia into macroreentrant and focal, the clinically relevant categories [20]. It is obvious that macroreentrant ATs are due to reentry but the electrophysiological mechanism of focal AT could be automaticity, triggered activity, or localized reentry. Importantly, in the era of AF ablation, majority of focal ATs seem to be due to localized reentry described here. Amongst the ATp, 46% to 75% represent macroreentrant ATs and 25% to 53% represent focal ATs. Amongst the focal ATp, 26% to 50% are due to discrete focal source and 50% to 74% represent localized variety [21, 22].

## 4. Mechanisms of ATp

ATp can occur intraprocedurally and/or postprocedurally (early or delayed). There is a mechanistic difference between the ATs occurring intraprocedurally and postprocedurally. It has been observed that the mechanism of ATp depends upon the clinical type of AF and the ablation strategy, to a great extent.

### 4.1. Intraprocedural ATp

Using a stepwise approach to ablation of longstanding AF, PV isolation is followed by electrogram-based atrial defragmentation. During this stage of ablation, usually, there is an increase in the AF cycle length (AFCL) compared to baseline AFCL. Often, at this very stage, AF converts into slower atrial tachycardia or flutter (CL ≥ 200 ms approximately). Thus, AT is observed as a transitional rhythm from AF to SR before the third and final step of linear ablation is undertaken [19]. Such intraprocedural ATp is suggestive of hidden organization in the disorganized appearing AF. When AF organizes into slow sustained AT, it is observed as a sign of impending restoration of SR which is eventually achieved on completion of linear and/or focal ablation. Spectral analysis of fibrillatory animal and human atria has shown results consistent with this transitional phenomenon observed in the clinical laboratory [23, 24].

### 4.2. Postprocedural ATp

After performing AF ablation, usually, variable degree of inflammatory state exists in the atria. It takes about 6–12 weeks to form a fibrous scar at the site of ablative lesion. Post-AF ablation, this is usually observed as a blanking period marked by transient but recurrent nonclinical atrial arrhythmias. As inflammation heals, inhomogeneous areas of nonuniform scar interspersed with healthy and/or partially damaged atrial tissue emerge [25]. Thus, atrium gets transformed into electrophysiologically heterogeneous chamber with uneven distribution of scar tissue. Areas of low voltage and slow conduction properties coexist as gaps amidst the nonconducting scar tissue generating a substrate highly favourable for reentrant arrhythmias. The likelihood of gaps increases with increase in the amount of tissue ablated. Arrhythmias observed beyond the blanking period are attributed to iatrogenically created proarrhythmic electrophysiology of extensively ablated atrial chamber.

In contradistinction to intraprocedural ATp which occur before linear ablation, most of the post procedural ATp develop after linear ablation of longstanding AF.

## 5. Locations of ATp

It has been observed that the volume of tissue ablation probably dictates the incidence of ATp. The volume of tissue ablated is directly related to the clinical type of AF. In paroxysmal AF, PV isolation is enough to cure AF in majority of the patients. Since the lesions are limited around PV ostium in the antrum, the PV ostia are the likely sites of focal ATp. Moreover, circumferential lesions involve higher volume of tissue than segmental lesions explaining a substantial contribution of the former to the rising incidence of ATp.

For longer lasting forms of AF, wherein circumferential PV isolation is combined with linear ablation in the roof, posterior left atrium or posterolateral mitral isthmus, macroreentry is established most likely around the mitral annulus or ipsilateral pulmonary venous antrum. Mitral annular and roof dependent flutters are the consequential ATp in the same order of frequency. Cavotricuspid isthmus (CTI) dependent ATp is another likely macroreentrant tachycardia although it is the least common variety.

**Figure 1:**
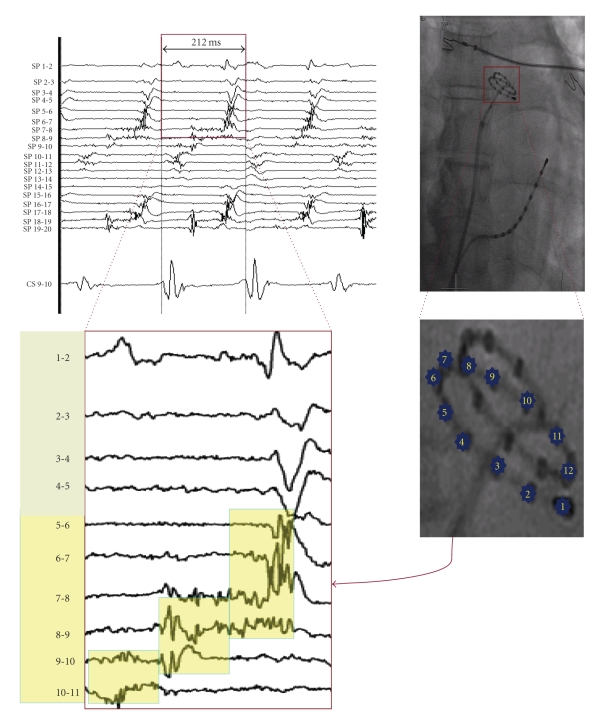
Cardiac fluoroscopy in posteroanterior view shows a multipolar spiral catheter (20 mm diameter) and a decapolar catheter. The spiral catheter is mapping left atrial roof. Within a small area of ~3 cm^2^, electrical activity spanning across almost the entire cycle length (212 ms) of tachycardia is demonstrated on bipolar electrograms recorded from dipoles 4-5 to 10-11 on the spiral catheter (yellow background in the zoom). The electrograms are low voltage with maximum amplitude −0.2 mV. This is characteristic of localized reentrant form of focal atrial tachycardia. Instead of a point source, small area harbours tiny circuit which sustains entire cycle length of reentrant tachycardia. The remainder of the atria is activated centrifugally which is consistent with focal tachycardia. Simultaneous recording of dipole 9-10 of decapolar catheter in coronary sinus is also displayed.

Localized circuits get established in small areas most commonly at the venous ostia (PV ostia, ostium of superior vena cava and coronary sinus), left septum, mouth of left atrial appendage (LAA), posterior left atrium and posterolateral mitral isthmus (ligament of Marshall). Such localized small circuits give rise to focal ATp from these sites. Left atrium (LA) adjoining anterior mitral annulus is an uncommon site of focal ATp [21, 22]. Unlike the point sources of typical focal AT, these sites involve small regions of slowly conducting tissue sustaining entire tachycardia circuit locally within <2 cm diameter (Figure 1). However, in concurrence with focal AT, the remainder of the atria gets activated centrifugally.

## 6. Diagnosis

### 6.1. Clinical Diagnosis

Clinically, the diagnostic and localizing value of surface ECG is debatable in atrial tachycardias arising after ablation. Since, the magnitude and direction of vector of atrial activation are tremendously influenced by the differential conduction velocity and low voltage areas of extensively ablated atria, surface P waves do not provide consistent information on the mechanism of ATp and the location of focal ATp. Therefore, regular ECG clues cannot be applied effectively to patients subjected to extensive ablation of the atria.

### 6.2. Electrophysiological Diagnosis

In the electrophysiology laboratory, it is important to diagnose the mechanism underlying ATp to achieve successful termination. As a preliminary step, it is important to acquire the information on previously ablated sites and determine if clear end point (bidirectional block) had been achieved. If patient is in SR during the repeat procedure, the evaluation for completeness of previously performed PV isolation should be the first step. If there is any evidence of PV reconnection, PV isolation should be completed before targeting other areas. If linear ablation involving CTI was performed previously, bidirectional block should be reconfirmed. Repeat ablation may be attempted to ascertain block.

**Figure 2:**
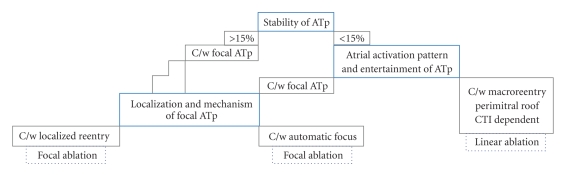
Stepwise Diagnostic Algorithm: A three-stepped approach to the diagnosis of atrial tachycardia during/post AF ablation (ATp). C/w—consistent with, CTI—cavotricuspid isthmus. (Modified from Jais et al. [22])

During ongoing tachycardia, we adopt and recommend a three-stepped approach (Figure 2) to diagnose the type of ATp based on the classification (macroreentrant versus focal) cited above [22]. We use a quadripolar irrigated tip mapping/ablation catheter in the atrial chamber and a decapolar catheter in the coronary sinus and implement the diagnostic approach during ongoing ATp as under.

**Step 1 (Determination of stability of ATp)**

Using the electrograms recorded from LAA and coronary sinus (CS) for one minute, the mean cycle length and the range of variation in cycle length over 1 minute is assessed. If the variation is >15% of the mean cycle length, focal mechanism is the most likely diagnosis. On the other hand, variation <15% does not rule out focal mechanism. In the latter situation, next step should be used to diagnose or rule out macroreentrant ATp.

**Step 2 (Determination of left atrial activation pattern and entrainment of ATp for Macroreentry)**

Considering that macroreentrant ATp would usually represent one of the following three: (1) mitral isthmus dependent flutter (Perimitral ATp), (2) roof dependent flutter, and (3) cavotricuspid isthmus dependent flutter, let us deductively infer each of these possibilities based on left atrial and CS activation patterns.

**Figure 3:**
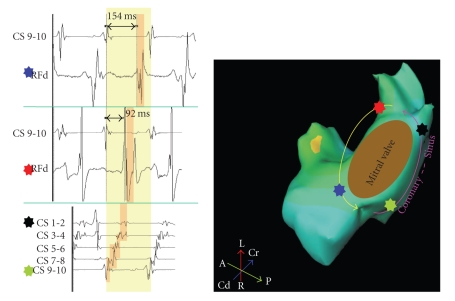
Atrial activation around the mitral annulus is shown during atrial tachycardia. Septal (green marker) to lateral (black marker) atrial activation pattern on decapolar coronary sinus (CS) catheter is demonstrated. Electrical activity recorded on mapping catheter (RF) in reference to proximal CS dipole 9-10 is demonstrated from two different sites (red and blue markers). Mapping catheter positioned at anterolateral mitral annulus (red marker) is activated 92 ms after proximal CS. Mapping catheter positioned at anteroseptal mitral annulus (blue marker) is activated 154 ms after proximal CS. Thus, during tachycardia, atrial activation proceeds laterally from the septum in the posterior left atrium and septally from the lateral aspect of anterior left atrium. This pattern conforms to that of perimitral flutter. Activation pattern around the mitral annulus comprises of the entire cycle length of this flutter (orange pillars march through yellow background which represents one cycle length).

**Figure 4:**
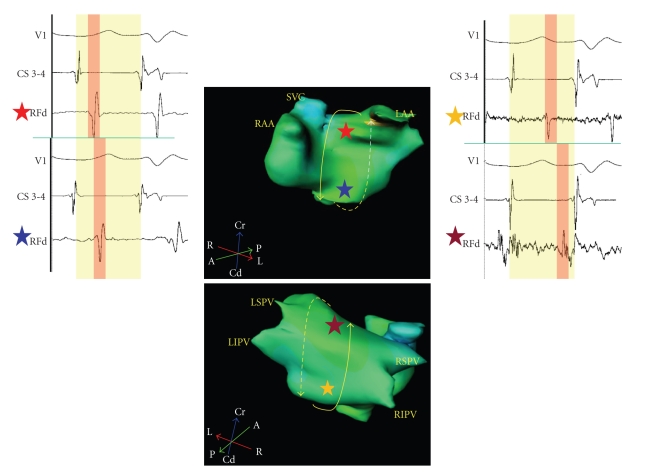
The electrogram recordings from four sites (red, blue, yellow, and maroon stars) on anterior and posterior left atrium are timed with coronary sinus dipole (CS 3-4) as reference. Local electrograms recorded from the high (red star) and low (blue star) anterior sites are 168 ms and 214 ms, respectively from the reference suggestive of high to low anterior left atrial activation pattern. Local electrograms recorded from high (maroon star) and low (yellow star) posterior sites are 274 ms and 262 ms, respectively from the reference suggestive of low to high posterior left atrial activation pattern. This pattern is consistent with roof dependent flutter.

- If the left atrial activation pattern is septal to lateral posteriorly and lateral to septal anteriorly (or vice versa) and the anterior and posterior walls activate low to high, counterclockwise (or clockwise) *perimitral ATp* is the most likely possibility (Figure 3).
- If the left atrial activation pattern is septal to lateral both anteriorly and posteriorly and the anterior and posterior walls activate high to low and low to high (or vice versa), respectively, *roof dependent ATp* rotating counterclockwise (or clockwise) around the antrum of *right PVs* is the most likely possibility. If the left atrial activation pattern is lateral to septal both anteriorly and posteriorly and the anterior and posterior walls activate low to high and high to low (or vice versa), respectively, *roof dependent ATp* rotating counterclockwise (or clockwise) around the antrum of *left PVs* is the most likely possibility. From therapeutic standpoint, we do not need to categorize roof dependent circuit as right or left (Figure 4).
- The left atrial activation pattern in typical counterclockwise *CTI dependent flutter* would be septal to lateral and low to high both on the anterior and posterior walls. In contrast, clockwise CTI dependent flutter would demonstrate high to low activation on the anterior and posterior walls of the left atrium.

Entrainment of ATp is a confirmatory step in the diagnosis of macroreentry. Importantly, if the ATp is entrained from three (at least two segments to avoid inadvertent termination or conversion to another tachycardia due to rapid pacing) distinct atrial segments (e.g., septal, posterior, and lateral LA for perimitral flutter) with postpacing interval (PPI) < 30 ms at each of the sites, macroreentry is confirmed. If this is not the case, macroreentry is ruled out and focal tachycardia is the only possible diagnosis.

**Step 3 (Localization of Focal ATp (Localized Reentry: A new variant))**

**Figure 5:**
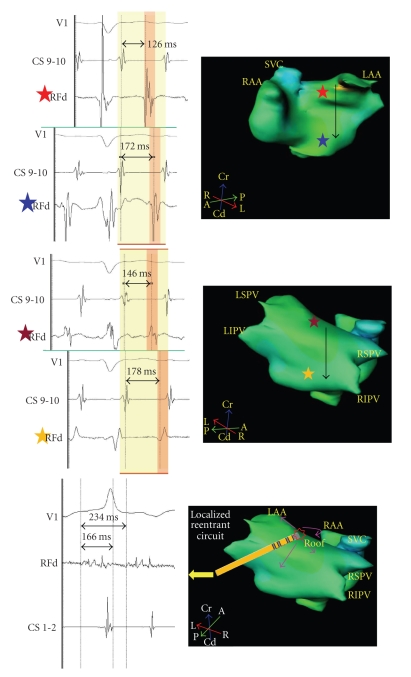
Top: The electrogram recordings from four sites (red, blue, yellow, and maroon stars) on anterior and posterior left atriums are timed with coronary sinus dipole (CS 9-10) as reference. Local electrograms recorded from high (red star) and low (blue star) anterior sites are 126 ms and 172 ms, respectively from the reference suggestive of high to low anterior left atrial activation pattern. Local electrograms recorded from the high (maroon star) and low (yellow star) posterior sites are 146 ms and 178 ms, respectively from the reference suggestive of high to low posterior left atrial activation pattern. This pattern which rules out macroreentry is consistent with focal tachycardia arising from a focus. Bottom: Focus is shown in the roof shown as “
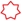
”. The arrows display centrifugal activation of the atria. Ablation catheter (RF) records continuous low voltage (0.176 mV) electrical activity spanning 166ms during atrial tachycardia (cycle length 234 ms). Recording 71% of tachycardia cycle length at a single spot suggests localized reentry as the mechanism of tachycardia.

The atrial activation pattern reflects the direction of centrifugal propagation of ATp from the focal source. In a single tachycardia cycle, the activation pattern of CS (proximal to distal or distal to proximal) and the left atrial activation pattern (based on local activation time of the anterior, posterior, septal, and lateral LA) which do not conform to macroreentrant mechanism provide a useful guide for mapping the focal source. Recording fragmented electrogram spanning over 50–75% of tachycardia cycle length is suggestive of site of localized small circuit (Figure 5).

The entrainment response for focal ATp is characteristically distinct from macroreentry. Of note, in contrast to macroreentrant circuit which is spread over more than one atrial segment, small circuit can be localized to one atrial segment only. On entrainment, PPI continues to get shorter as pacing site gets closer to the focus. This phenomenon provides an important guide to segmental localization of focal ATp [26]. PPI < 30 ms suggests that the pacing site is near/at the site of interest. Sometimes, it is difficult to establish capture at the site of interest even with high pacing output. In such a situation, pacing from an area in proximity to the site of interest would yield PPI in the range of 30 ms to 50 ms, which is acceptable. Entrainment farther from the proxy site yields PPI > 50 ms. Thus shorter the PPI, closer the mapping catheter to tachycardia source. If the segment of interest cannot be localized to the LA based on the ATp activation pattern and entrainment criterion, right atrial mapping is performed in the same manner.

In contrast to typical focal ATs arising in normal hearts, focal ATp demonstrating automatic mechanism of initiation and maintenance of tachycardia is less commonly encountered in post-AF ablation patients. High variability in AT cycle length is characteristically described for automatic tachycardias with spontaneous onset and offset with gradual warm up and decline, respectively. The centrifugal atrial activation pattern can be helpful in mapping the source of the focus. Instead of continuous low voltage fragmented local activity on electrograms, a sharp and high voltage presystolic potential with high negative dV/dT monophasic (QS shaped) unipolar electrogram will be obtained at the site of origin of AT. Entrainment of automatic ATs is not feasible. Overdrive pacing of automatic tachycardias will have a variable response from the same site of pacing. Postoverdrive pacing recovery time of automatic focus is highly variable. As against it, fixed postpacing interval can be obtained after entrainment of focal AT due to localized reentry.

### 6.3. Three Dimensional Mapping Tools

Three dimensional electroanatomical mapping systems are not indispensable for diagnosis and ablation of ATp. For those who are conversant with the fluoroscopic anatomy of atria and their venous inputs and perform electrogram guided ablation of ATp expeditiously, 3D systems do not offer any specific advantage in majority of the cases. Very often, more than one ATp sequentially occurs before restoration of SR. Conversion of target tachycardia to another tachycardia will entail reconstruction of activation maps every time it happens. Besides, after building the maps, entrainment maneuvers cannot be dispensed with for establishing and/or confirming the diagnosis. Moreover, critical areas of low amplitude signals may be ignored as noise. This can cause disparity between clinical tachycardia and reconstructed electroanatomical map especially for localized reentrant ATp. Though it is a matter of operator preference, 3D systems are not essential for ATp ablation. Instantaneous multielectrode epicardial mapping systems have the advantage of building atrial activation maps within 8–10 minutes noninvasively. Sustenance of tachycardia is not mandatory. Preprocedural diagnosis regarding the mechanism and source of ATp may be obtained noninvasively. However, clinical utility in this area will require validation.

## 7. Drug Therapy

Usually, AF ablation is undertaken in patients who fail to respond to drug therapy. The response to drug therapy may improve postablation in patients with recurrent AF. However, ventricular rate gets faster during ATp than AF. Often, ATp is very stable and sustained. Patient tolerance is worse for ATp than AF for such reasons. Though there are no randomized trials involving drug therapy in ATp, it is observed that ATp do not effectively respond to the drugs. On the other hand, ablation offers cure for the arrhythmia. Consequentially, catheter ablation is preferred over medical management for ATp.

## 8. Catheter Ablation

The ablation of focal ATp is accomplished more easily than the diagnosis. The reverse holds true for macroreentrant ATp. Successful termination of target tachycardia is the common endpoint for both the types of ATp. Conversion of target tachycardia to another tachycardia with same/different cycle length and different atrial activation pattern is frequently encountered before eventual restoration of SR. Therefore, close monitoring of electrograms during tachycardia ablation is very important. Finer changes in activation pattern and cycle length may be the only subtle clues towards requirement of repeating diagnostic maneuvers including the entrainment response.

Focal ATp are ablated at the site of origin. Using saline irrigated tip catheter and a target temperature upto 42°C, radiofrequency energy with power of upto 30 W can be delivered for 30–60 seconds at each point with continuous monitoring of electrograms. Transient acceleration of tachycardia following the commencement of energy-delivery may occur when ablation is localized at the right spot. If tachycardia fails to terminate or convert to another tachycardia after at least 60 s of energy delivery, further mapping is performed to localize a better site for ablation. 

Macroreentrant ATp are targeted by performing linear ablation. CTI ablation is performed conventionally for CTI dependent flutters. Linear ablation of the left atrial roof is performed by joining the ostia of two superior pulmonary veins at the most cranial aspect of LA. It terminates both the right and the left sided roof dependent flutter. Perimitral flutter is targeted by performing linear ablation at the posterolateral mitral isthmus. Ablation of mitral isthmus is performed by withdrawing the catheter from the ventricular side of the mitral isthmus (where the atrial to ventricular electrogram ratio is 1 : 1 or 2 : 1) to the left inferior PV. Ablation within the CS on the epicardial side of the mitral isthmus is performed when bidirectional conduction block cannot be achieved across the isthmus after at least 20 minutes of endocardial energy application. Power of up to 35 W endocardially and upto 25 W epicardially is required to achieve transmural lesion in most resistant cases. The endpoint is creation of line of complete block. Confirmation of bidirectional block is undertaken following restoration of SR.

**Figure 6:**
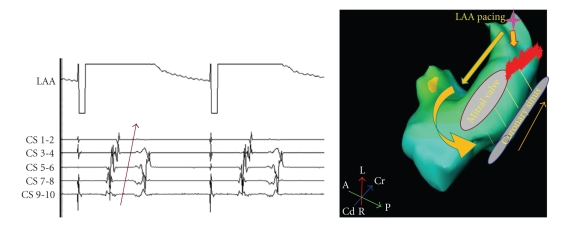
Left atrial appendage (LAA) pacing (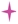) results in proximal to distal activation (arrow) of coronary sinus (CS). This is suggestive of blocked (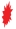) posterolateral mitral isthmus, at least, unidirectionally. CS 9-10—proximal CS, CS 1-2—distal CS.

Block across the mitral isthmus linear ablation is demonstrated by differential pacing from distal and proximal CS dipoles sequentially and recording the time of activation in LAA. If LAA activation is earlier with proximal CS pacing than more distal CS pacing, it is suggestive of unidirectional block. If subsequent pacing from LAA shows that the activation in CS proceeds from proximal dipole distally, it is conclusive of bidirectional block (Figure 6). Estimation of block across the roof linear ablation is undertaken in SR or LAA pacing. If the activation of posterior LA occurs low to high, it confirms unidirectional block along the roof line. Subsequent differential pacing on the posterior LA and recording the time of activation in LAA is done to confirm bidirectional block. LAA activation with inferoposterior LA pacing should occur earlier than superoposterior LA pacing to conclude that the roof linear ablation is bidirectionally blocked. Presence of widely split double-potentials across the line of block reinforces the completeness of linear ablation [7, 27].

## 9. Procedural Outcome and Prognosis

Using the algorithm mentioned above, 97% ATp were diagnosed in less than 11 minutes of mapping duration in a recently published report from our center. Incoherent mapping due to widespread areas of altered electrogram characteristics/scars and noninducibility after inadvertent termination of tachycardia caused difficulty in diagnosis. All the diagnosed cases were treated successfully. Complete line of block at mitral isthmus, left atrial roof and CTI was achieved in 95%, 97%, and 100% cases, respectively. At follow-up of 21 ± 10 months, recurrence of sustained ATp was observed in 5%. After repeat ablation procedure, SR was maintained in 95% patients [22].

## 10. Prevention of ATp

It is proven beyond doubt that ATp is partly an iatrogenic proarrhythmia. Since the volume of ablation drives the probability of occurrence of ATp, limiting the ablation within the atrium can help reduce the incidence of certain forms of ATp. Judicious use of ablative energy can help minimize the incidence of reentrant and focal ATp. Macroreentrant ATp develop frequently in patients with incompletely blocked previous linear ablation. Ensuring that bidirectional block is achieved and documented after linear ablation can prevent recurrent macroreentry. An important lesson learnt from AF ablation procedures is that application of energy unscrupulously and indiscriminately is not helping cure AF. Therefore, limiting ablation to achieve isolation of PVs in paroxysmal AF and PV isolation plus necessary defragmentation and linear ablation in persistent and long lasting forms of AF can retard the epidemically growing pace of ATp.

## 11. Conclusion

In the era of AF ablation, there is an increase in the incidence of both macroreentrant and focal ATs. Extensive substrate modification generates focal areas of slow conduction and low voltage capable of sustaining localized reentry, a novel mechanism of reentrant atrial arrhythmias post-AF ablation. ATp is also the final common pathway towards restoration of SR in catheter ablation of long lasting AF. Ascertaining bidirectional block after linear ablation and minimizing the volume of tissue ablation can help reduce the incidence of ATp. Using conventional tools and electrogram guidance for catheter ablation, deductive algorithm presented here can meticulously diagnose and cure ATp.
